# Genomic ancestry and ethnoracial self-classification based on 5,871 community-dwelling Brazilians (The Epigen Initiative)

**DOI:** 10.1038/srep09812

**Published:** 2015-04-27

**Authors:** M. Fernanda Lima-Costa, Laura C. Rodrigues, Maurício L. Barreto, Mateus Gouveia, Bernardo L. Horta, Juliana Mambrini, Fernanda S. G. Kehdy, Alexandre Pereira, Fernanda Rodrigues-Soares, Cesar G. Victora, Eduardo Tarazona-Santos, Epigen-Brazil group, Cibele C. Cesar, Jackson S. Conceição, Gustavo N.O. Costa, Nubia Esteban, Rosemeire L. Fiaccone, Camila A. Figueiredo, Josélia O.A. Firmo, Andrea R.V.R. Horimoto, Thiago P. Leal, Moara Machado, Wagner C.S. Magalhães, Isabel Oliveira de Oliveira, Sérgio V. Peixoto, Maíra R. Rodrigues, Hadassa C. Santos, Thiago M. Silva

**Affiliations:** 1 Fundação Oswaldo Cruz, Instituto de Pesquisas Rene Rachou, Belo Horizonte, Brazil; 2 London School of Hygiene and Tropical Medicine, Department of Infectious Disease Epidemiology, London, UK; 3 Universidade Federal da Bahia, Instituto de Saúde Coletiva, Salvador, Brazil; 4 Universidade Federal de Minas Gerais, Instituto de Ciências Biológicas, Belo Horizonte, Brazil; 5 Universidade Federal de Pelotas, Programa de Pós Graduação em Epidemiologia, Pelotas, Brazil; 6 Universidade de São Paulo, Instituto do Coração, São Paulo, Brazil; 7 Universidade Federal de Minas Gerais, Instituto de Ciências Exatas, Belo Horizonte, Brazil; 8 Universidade Federal da Bahia, Instituto de Saúde Coletiva, Salvador, Brazil; 9 Universidade de São Paulo, Instituto do Coração, São Paulo, Brazil; 10 Fundação Oswaldo Cruz, Instituto de Pesquisas Rene Rachou, Belo Horizonte, Brazil; 11 Universidade Federal de Minas Gerais, Instituto de Ciências Exatas, Belo Horizonte, Brazil

## Abstract

Brazil never had segregation laws defining membership of an ethnoracial group. Thus, the composition of the Brazilian population is mixed, and its ethnoracial classification is complex. Previous studies showed conflicting results on the correlation between genome ancestry and ethnoracial classification in Brazilians. We used 370,539 Single Nucleotide Polymorphisms to quantify this correlation in 5,851 community-dwelling individuals in the South (Pelotas), Southeast (Bambui) and Northeast (Salvador) Brazil. European ancestry was predominant in Pelotas and Bambui (median = 85.3% and 83.8%, respectively). African ancestry was highest in Salvador (median = 50.5%). The strength of the association between the phenotype and median proportion of African ancestry varied largely across populations, with pseudo R^2^ values of 0.50 in Pelotas, 0.22 in Bambui and 0.13 in Salvador. The continuous proportion of African genomic ancestry showed a significant S-shape positive association with self-reported Blacks in the three sites, and the reverse trend was found for self reported Whites, with most consistent classifications in the extremes of the high and low proportion of African ancestry. In self-classified Mixed individuals, the predicted probability of having African ancestry was bell-shaped. Our results support the view that ethnoracial self-classification is affected by both genome ancestry and non-biological factors.

Brazil is the 5^th^ most populous nation in the world, with about 200 million inhabitants^1^. Its population originated from three main ancestral roots: African, European and Native American, the latter constituting the autochthonous population. Colonization was predominantly Portuguese. The slave trade of Africans to Brazil was the oldest, the longest-running and the largest in the Americas. Early European colonizers and their descendants brought an estimated of 3.6 million African slaves, seven times more than their counterparts in the United States^2^.

Brazil never had segregation laws defining who should belong to an ethnoracial group, as the United States and South Africa had. This was probably a result of the Brazilian elite decision to “whiten” the Brazilian population through miscegenation rather than impose segregation; and ethnoracial classification was left to individual perception^2^. As a consequence, the composition of the Brazilian population is more mixed, and its ethnoracial classification is more complex and fluid than in those countries where segregation was imposed by law^2^. This was to such a degree that it has been questioned whether – and how – ethnoracial classification in Brazil correlates with genomic ancestry. Previous genome studies based on up to a hundred informative markers showed conflicting results on this correlation^3^,^4^,^5^,^6^,^7^,^8^.

The Brazilian census adopts a classification based on ethnoracial self-classification with five groups: White, Mixed (“pardo” in official Portuguese), Black, Yellow (Asian) and Indigenous (Native American), the latter two representing less than 1% of the total population^1^. People who self-report as Whites predominate in the South and Southeast, and as Mixed and/or Black in the North and Northeast^1^. Persons self-reporting as Black and Mixed are more likely to have lower income and education^2^,^9^,^10^,^11^, to report experiencing discrimination^11^,^12^, and have more negative health-related outcomes^11^,^13^,^14^,^15^,^16^,^17^. The most plausible explanation for these disparities is the cumulative effect of the lack of social policies to support individuals of African origin and their descendants since the abolition of slavery in 1888^18^. To some extent, recent affirmative action in Brazil, mostly based on ethnoracial self-classification, is supported by this theoretical framework. Thus, the debate over whether ethnoracial self-classification correlates with ancestry has scientific and policy implications.

The Epigen-Brazil initiative is based on three well-defined ongoing population-based cohorts from Brazil’s South^19^, Southeast^20^ and Northeast^21^. We used 370,539 Single Nucleotide Polymorphisms (SNPs) to quantify the association between likelihood of self-classification as White, Mixed and Black and genome-wide based individual proportions of African, European and Native American ancestry in 5,851 participants of these cohorts.

## Results

The study included 3,533 individuals from Pelotas (South), 1,442 from Bambuí (Southeast), and 876 from Salvador (Northeast). Self-reported as White predominated in Pelotas (77.5%) and Bambuí (60.6%), while self-reported as Black (43.4%) and Mixed (49.3%) predominated in Salvador. The Pelotas and the Bambuí cohort populations had predominant European ancestry (median = 85.3% and 83.8%, respectively), while African ancestry was the highest in Salvador (median = 50.5%). Native American ancestry was little and relatively uniform in the three sites (~ 5-6%) (Table 1).

**Figure 1:**
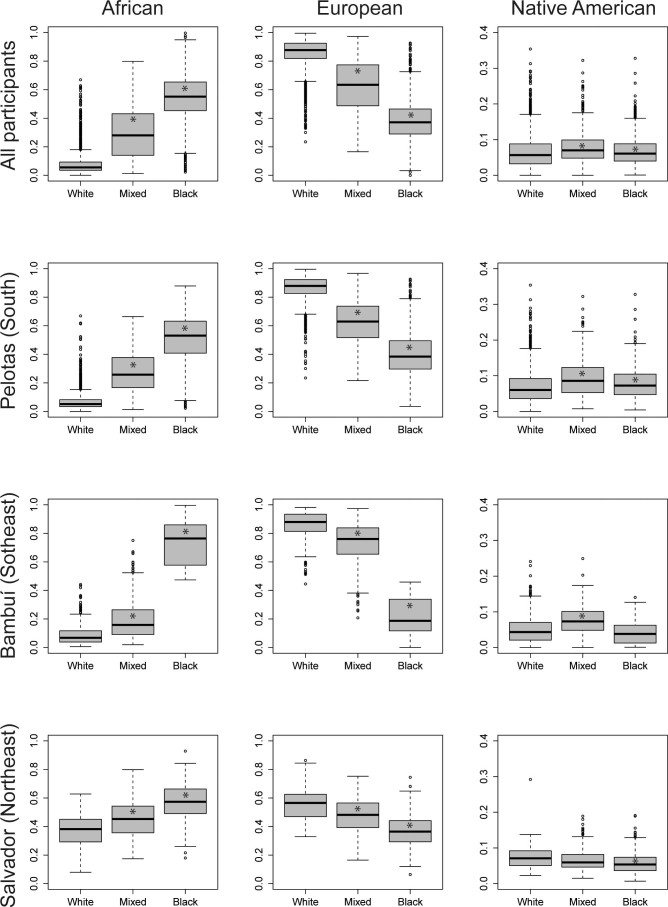
Box plot contrasting ethnoracial self-classification (White, Mixed and Black) to median individual proportion of genomic African, European and Native American ancestries in all participants, and by cohort population (The Epigen Initiative). Mixed is “pardo” in official Portuguese. (*) p <0,001 for comparisons between each ethnoracial category to White.

Median African, European and Native American individual ancestry across ethnoracial categories are shown in 12 panels in Figure 1. In the joint analysis of the 3 cohorts, as well as within each cohort population, there was a significant increase on the median African ancestry from people self-reporting as White to Mixed and then to Black (p<0.001 in Mann Whitney test for differences across ethnoracial categories); median European ancestry decreased in the opposite direction, as expected. It is of note, however, that the distribution of African and European ancestry across ethnoracial categories showed more overlapping in Salvador than in the other sites. With regards to Native American ancestry, there was no clear pattern: in Pelotas, persons self-reported as Mixed and Black had significant higher median of Native American ancestry than Whites; in Bambuí, only persons self-reporting as Mixed showed higher level of Native ancestry, while in Salvador this was true only for persons self-reporting as White.

Ethnoracial self-classification as White, Mixed and Black in each cohort, by quartiles of individual African ancestry are shown in Table 2. Self-reporting as Black were more likely at the highest quartile of African ancestry in Pelotas (83.8%), Bambui (100.0%) and Salvador (97.2%). In contrast, we found a stronger likelyhood of self reporting as White at the highest quartile of African ancestry in Salvador (60.0%) relative to Pelotas (0.7%) and Bambui (0.8%). Results of the quantile regression anlysis showed that the strength of the association between the phenotype and African ancestry varied largely across the 3 sites, with pseudo R^2^ values of 0.50 in Pelotas, 0.22 in Bambui and 0.13 in Salvador in the analysis comparing those above/bellow median of African ancestry. The differences across populations remained in the analyses comparing those above/below the 0.75 percentile of African ancestry (pseudo R^2^ = 0.64, 0.32 and 0.13, respectively).

**Figure 2:**
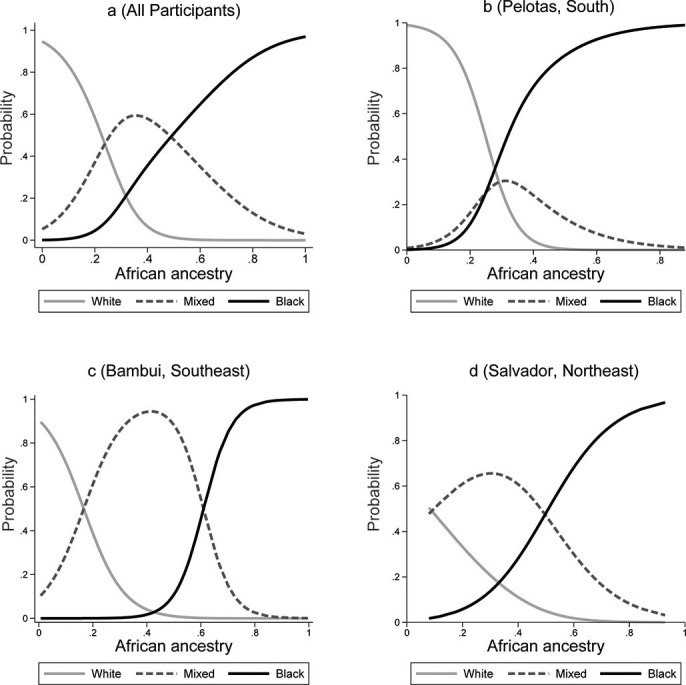
Predicted probability of ethnoracial self-classification as Black, Mixed and White along the genomic proportion of African ancestry continuum in all participants, and by cohort population (Epigen-Brazil). Mixed is “pardo” in official Portuguese.

The joint analysis and the analysis by cohort population of the predicted probabilities of self-reporting as Black, Mixed and White along the African ancestry continuum are shown in Figure 2. African genomic ancestry showed an S-shape positive association with self-reporting as Black, which was consistent in all populations, whereas the reverse was observed for self-reporting as White. In the joint analysis, as well as for each cohort separately, these trends were statistically significant (p<0.001 in Wald́s test). The probability of self-reporting as Black increased sharply as the proportion of African ancestry reached about 20% in Pelotas and 40% in Bambuí. The probability of self-reporting as White decreased sharply as the proportion of African ancestry reached about 10%-20% in these two populations. These increase/decrease were smoother in Salvador than in the other two sites. Self-classified Mixed individuals showed a bell-shaped predicted probability of having African ancestry in all sites.

## Discussion

This is the first large community-based multicenter study to investigate the association between individual proportions of genome-wide based African, European and Native American ancestries and likelihood of ethnoracial self-classification in Brazil. The key findings are: first, the association between the phenotype and genome ancestry was statistically significant, but the strength of the association varied largely across populations; second: the association between Black and White self-classification with ancestry was most consistent in the extremes of the high and low proportion of African ancestry.

We confirmed previous historical and genetics reports of the largest African ancestry observed in Northeastern, as well as predominant European ancestry in Southeastern and Southern Brazil^2^,^5^,^7^,^22^. Furthermore, the contribution by Native Americans to the studied individuals was consistently small in the three sites. This is also in agreement with genetic reports indicating that Native American ancestry is higher in the North-West Brazil (Amazonia), a region that was not considered in our analysis^7^.

In order to examine whether – and how – ethnoracial classification correlates with genomic ancestry, we used three different methods of analyses. The first (a population measure), aimed at assessing how ethonoracial self-classification varied by medians of African, European and Native American ancestry. The other two methods, based on individual level measures, aimed at comparing the likelihood of the self-classification at the same levels of African ancestry across populations, as well as assessing how the relationship between ethnoracial self-classification changed along the proportion of genomic African ancestry continuum. Our results showed statistically significant associations between ancestry and the phenotype both at population and individual levels. However, the extent of overlap of individual proportions of each ancestry across ethnoracial groups was more evident in the Salvador population relative to the other sites. The association between Black and White self-identification with African ancestry continuum scale was S shape in all sites, but smoother in the Salvador population. Further, those who self-identified as Mixed tended to show intermediate proportions of African ancestry in all studied populations. This is in agreement with sociological and demographic conceptions that Mixed (“pardo” in official Portuguese) comprises multiple terms of popular discourse denoting ethnoracial admixture in Brazil^2^.

Previous sociological studies have suggested that ethnoracial self-classification in Brazil may tend to avoid nonwhite, and especially Black, categories since these were often associated with negative characteristics^2^. They suggest that miscegenation tends to shift self-reporting towards White, while segregation – as in the United State – would tend to shift self-reporting towards Black^2^. Our results indicate that avoidance of Black category may not be generalizable for the Brazilian population. In the current study, this effect appears to happen only in individuals from Salvador, where persons at the highest proportion of African ancestry were more likely to call themselves White relative to their counterparts from Pelotas and Bambui.

This study has strengths and limitations. Strengths include the very large number of SNPs used and the use of large community-based samples from different regions of eastern Brazil, as well as the fact that, the same set of reference populations (representing European, African, and Native American individuals) have been used in analyzing the three cohorts; thus, the inferred admixture ratios are comparable among the studied populations. Although the Pelotas and Bambuí cohorts are representative of the general population of their respective areas, in the eligible age groups, the cohort in Salvador oversampled individuals living in poor environments; thus, although there is good internal consistency, the results cannot be interpreted as representing the whole population of this city.

Summarizing, our results respond to three main sociological questions^2^ that were not answered yet. They are: first, ethnoracial self- classification in Brazilians is certainly not random with respect to genome individual ancestry; second, the association between ethnoracial self-classification and genome based ancestry is not linear, with most consistent associations in the extremes of the African ancestry continuum scale; third, a tendency to whitening ethnoracial self-identification was found in persons from Salvador (where African ancestry is more common), but not in persons from the remaining two sites (where European ancestry predominates). Our results provides support to the view that ethnoracial self-classification is affected by both genomic ancestry and non-biological factors.

## Methods

### Cohort designs and ethnoracial self-classification

The 1982 Pelotas birth cohort study was conducted in Pelotas, a city in Brazil’s extreme South, near the Uruguay border, with 214 000 urban inhabitants in 1982. Throughout 1982, the three maternity hospitals in the city were visited daily and births were recorded, corresponding to 99.2% of all births in the city. The 5,914 live-born infants whose families lived in the urban area constituted the original cohort. At age of 23 years, 3,736 participants categorized themselves according to the five ethnoracial categories used by the Brazilian census^1^, as previously described. The Native American and yellow categories (67 and 64 individuals, respectively) were excluded from the current analyses. Further details are shown in a previous publication^19^.

The Bambui cohort study of ageing is ongoing in Bambuí, a city of approximately 15,000 inhabitants, in Minas Gerais State in Southeast Brazil. The population eligible for the cohort study consisted of all residents aged 60 years and over on 1 January 1997, who were identified from a complete census in the city. Of a total of 1,742 older residents, 1,606 constituted the original cohort. At baseline, 1,442 participants categorized themselves into the above mentioned ethnoracial groups^1^, according to standard photographs of Brazilians; no individuals categorized themselves as Amerindian or yellow. Further details of the Bambui study can be seen elsewhere^20^.

The Salvador-SCAALA project is a longitudinal study involving a sample of 1,445 children aged 4-11 years in 2005, living in Salvador, a city of 2.7 million inhabitants in Northeast Brazil. The population is part of an earlier observational study that evaluated the impact of sanitation on diarrhea in 24 small sentinel-areas selected to represent the population without sanitation in Salvador. In the 2013 follow-up, 879 participants categorized themselves according to the previous mentioned ethnoracial groups^1^ and were included in the present analysis; in the same way as in Bambui, no individuals categorized themselves as Amerindian or yellow in Salvador. Further details can be seen elsewhere^21^.

### Genotyping and external parental populations

The Epigen-Brazil participants were genotyped by the Illumina facility (San Diego, California) using the Omni 2.5M array. We performed the unsupervised tri hybrid (k = 3) ADMIXTURE analyses based on 370,539 SNPs shared by samples from the HapMap Project, the Human Genome Diversity Project (HGDP)^23^,^24^and the Epigen-Brazil study population. As external panels, we used the following HapMap samples: 266 Africans (176 Yoruba in Ibadan, Nigeria [YRI] and 90 Luhya in Webuye, Kenya [LWK]), 262 Europeans (174 Utah residents with Northern and Western European ancestry [CEU] and 88 from Toscans from Italy [TSI]), 170 admixed individuals (77 Mexicans from Los Angeles, California [MEX] and 83 Afro-African from Southwest USA [ASW]), and 93 Native Americans from the HGDP (25 Pima, 22 Karitiana, 25 Maya and 21 Surui). The same set of reference populations was used in analyzing the three cohorts.

### Family structure

To assess the familial structure, we estimated kinship coefficients for each possible pair of individuals from each cohort, using the method implemented in the REAP software (Related Estimation in Admixed Populations)^25^. This method was specifically developed to obtain accurate estimations of kinship coefficients in admixed populations, solely using genetic data and without using pedigree information. We considered a pair of individuals as related if the estimated kinship coefficient between them was ≥ 0.1. This cutoff includes second- degree relatives such as a person’s uncle/aunt, nephew/niece, grandparent/grandchild or half- sibling, and any closer pair of relatives. Based on this cut-off, we identified set of related individuals (i.e. families) and assigned to each individual a categorical variable that represent his/her family. Because Pelotas and Salvador showed very few families, we decided to exclude related individuals (defined on the basis of the above mentioned cut-off). Therefore, 72 persons from Pelotas and 3 from Salvador were excluded from this analysis because they were related. The Bambuí cohort participants showed an important family structure (885 were related), so excluding them would lead to loss of power and possibly a degree of selection bias, so we opted for keeping related individuals, and undertaking sensitivity analysis to assess the influence of family structure on our results.

### Statistical analyses

To take into account the differences across populations, we stratified analyses into the three study areas. To estimate the contribution from Africans, Europeans and Native Americans to the Epigen individuals we used the ADMIXTURE software^26^. We assumed three clusters to mimic the three main components of Brazilian ancestry, and used an unsupervised mode in order to allow the program to identify clusters corresponding to the ancestral populations solely from the genetic structure of our dataset. ADMIXTURE performs a model-based maximum-likelihood estimate of individual ancestry proportions, using an algorithm based on a sequential quadratic programming for block updates, coupled with a novel quasi-Newton acceleration of convergence.

Because the distribution of ancestry proportions was asymmetric, we calculated medians instead of means. Pearson’s chi-square test was used to assess statistical significance among frequencies, and Kruskal-Wallis rank test or Mann-Whitney test were used to assess statistical significance of differences among medians, respectively. We compared likelihood of individual self-ethnoracial classification at the same level of African ancestry. We examined this by examining proportions of White, Mixed and Black self-classification by quartiles of African ancestry, calculated for the population as a whole, including the people from the 3 cohorts. Quantile (median and 0.75) regression was used to estimate the strength of these associations^28^.

To quantify how the relationship between ethnoracial self-classification changed along the proportion of genomic African ancestry continuum, we fitted a multinomial logistic regression for the joint analysis of the three populations, adjusted for the cohort effect, and plotted the predicted probabilities for the outcome. Similar analyses were performed separated for each cohort population. A generalized Hosmer-Lemenshow goodness-of-fit test was use to assess the adequacy of the above mentioned multinomial models^27^^.^

For the Bambuí cohort, we did a sensitivity analyses to assess the influence of familial structure on our results. We verified this by examining the previous mentioned unadjusted multinomial models relative to a model containing a random effect term for adjustments for family structure^29^, and verified that this did not affect our results (not shown). Thus, our analysis were based on all Bambui cohort participants, irrespective of kinship.

The analyses were carried out for pooled men and women, given that in all populations sex showed no statistically significant associations with either ethnoracial classification or genetic ancestry. Furthermore, we excluded age from our analyses for two reasons: first, age distributions were homogeneous in the Pelotas and Salvador cohorts (23 years and 12-22 years, respectively); and, second, age showed no significant associations with ethnoracial self-classification, as well as with genomic ancestry, in the Bambui cohort population, whose age ranged from 60 to 95 years.

Statistical analyses were conducted using STATA 13.0 statistical software (Stata Corporation, College Station, Texas). All p-values were 2-tailed (alpha = 0.05).

### Ethics assessment

The Epigen protocol was approved by Brazil’s national research ethics committee (CONEP, resolution number 15895, Brasília). The research has been conducted according to the principles expressed in the Declaration of Helsinki. Participants signed an informed consent form and authorized their genotyping.

## Figures and Tables

**Table 1:** Ethnoracial self-classification and median individual proportion of African, European and Native American ancestries in all participants and by cohort population (Epigen-Brazil). (*) P <0.001 for differences across population. Mixed is “pardo” in official Portuguese.

	Cohort population			
	Pelotas (South) N = 3,533	Bambui (Southeast) N = 1,442	Salvador (Northeast) N = 876	AllN = 5,851
Ethnoracial classification, %				
Black	16.6	2.5	49.3 *	18.1
Mixed (“pardo”)^1^	5.8	36.9	43.3	19.1
White	77.5	60.6	7.4	62.9
Genomic ancestry, median (interquartile range)				
African	6.6 (3.8-16.3)	9.6 (4.8-17.5)	50.5 (40.9-60.4) *	9.2 (4.5, 33.8)
European	85.3 (72.8-91.0)	83.8 (74.2-91.2)	42.4 (33.7-52.3) *	82.1 (57.1, 90.1)
Native American	6.3 (3.8-9.6)	5.4 (2.8-8.4)	5.8 (4.2-7.8) *	6.0 (3.7, 9.0)

**Table 2:** Ethnoracial self-classification by quartiles of individual African ancestry, and by cohort population (Epigen-Brazil). B (95% CI): coeficient and 95% confidence intervals estimated by quantile regression. (*) p<0.01; (**) p<0.001. Mixed is “pardo” in official Portuguese.

	Quartiles									
	Total N	Lowest N(%)	2^nd^N (%)	3rd N (%)	Highest N (%)	B (95% CI)(median regression model)	B (95% CI)(0.75 regression model)			
**Pelotas (South)**							
White	2739	41.1	39.2	19.0	0.7	1.0	1.0
Mixed (“pardo”)^1^	206	3.4	5.3	58.7	32.5	0.21 (0.20. 0.23) **	0.30 (0.28, 0.31) **
Black	588	0.7	1.4	14.1	83.8	0.48 (0.47, 0.49) **	0.55 (0.54, 0.56) **
						Pseudo R^2^ = 0.50	Pseudo R^2^ = 0.64
**Bambui (Southest)**							
White	874	33.4	30.2	35.6	0.8	1.0	1.0
Mixed (“pardo”)	532	6.4	19.7	59.2	14.7	0.09 (0.08. 10.3) **	0.15 (0.13, 0.16) **
Black	36	0	0	0	100.0	0.70 (0.66, 0.74) **	0.73 (0.68, 0.79) **
						Pseudo R^2^ = 0.22	Pseudo R^2^ = 0.32
**Salvador (Northeast)**							
White	65	0	1.5	38.5	60.0	1.0	1.0
Mixed (“pardo”)^1^	379	0	0	19.8	80.2	0.07 (0.03, 11.5) *	0.09 (0.05, 0.13) **
Black	432	0	0	2.8	97.2	0.19 (0.15, 0.23) **	0.21 (0.17, 0.25) **
						Pseudo R^2^ = 0.13	Pseudo R^2^ = 0.13
